# Differentiate responses of soil nutrient levels and enzymatic activities to freeze-thawing cycles in different layers of moss-dominated biocrusts in a temperate desert

**DOI:** 10.3389/fpls.2023.1137754

**Published:** 2023-03-06

**Authors:** Qing Zhang, Jiwen Li, Shujun Zhang, Yonggang Li, Nan Wu, Xiaobing Zhou, Benfeng Yin, Yuanming Zhang

**Affiliations:** ^1^ State Key Laboratory of Desert and Oasis Ecology, Xinjiang Institute of Ecology and Geography, Chinese Academy of Sciences, Urumqi, China; ^2^ Key Laboratory of Crop Nutrition and Fertilization, Ministry of Agricultural/Institute of Agricultural Resources and Regional Planning, Chinese Academy of Agricultural Sciences, Beijing, China; ^3^ College of Life Science and Technology, Xinjiang University, Urumqi, China; ^4^ Yantai Key Laboratory of Coastal Hydrological Processes and Environmental Security, Ludong University, Yantai, Shandong, China

**Keywords:** climate change, biological soil crusts, soil nutrient multifunctionality, biogeochemical cycles, temperate deserts

## Abstract

**Introduction:**

The biological soil crust, a widespread phenomenon in arid and semi-arid regions, influences many ecological functions, such as soil stability, surface hydrology, and biogeochemical cycling. Global climate change has significantly altered winter and spring freeze-thaw cycles (FTCs) in mid and high-latitude deserts. However, it is unclear how these changes will affect the biological soil crust and its influence on nutrient cycling and soil enzyme activity.

**Methods:**

We conducted this study in the Gurbantunggut Desert, a typical temperate desert, using the moss crust as an example of an evolved biological soil crust. Simulating the effects of different FTC frequencies (0, 5, and 15 times) on soil carbon, nitrogen, phosphorus-related nutrients, and extracellular enzyme activities allowed us to understand the relationship between soil environmental factors and nutrient multifunctionality during FTC changes.

**Results:**

The results showed that recurrent FTCs significantly increased the accumulation of carbon and phosphorus nutrients in the soil and decreased the effectiveness of nitrogen nutrients. These changes gradually stabilized after 15 FTCs, with available nutrients showing greater sensitivity than the previous full nutrient level. FTCs inhibited carbon, nitrogen, and phosphorus cycle-related hydrolase activities and promoted carbon cycle-related oxidase activities in the crust layer. However, in the 0–3 cm layer, the carbon and phosphorus cycle-related hydrolase activities increased, while peroxidase and urease activities decreased. Overall, the nutrient contents and enzyme activities associated with the carbon, nitrogen, and phosphorus cycles were lower in the 0–3 cm layer than in the crust layer. In addition, the multifunctionality of nutrients in the soil decreased after 15 FTCs in the crust layer and increased after 5 FTCs in the 0–3 cm layer. Structural equation modeling showed that FTC, soil water content, pH, available nutrients, and extracellular enzyme activity had opposite effects on nutrient multifunctionality in different soil layers. The change in nutrient multifunctionality in the crust layer was primarily caused by changes in total nutrients, while soil water content played a greater role in the 0–3 cm layer. Regardless of the soil layer, the contribution of total nutrients was much higher than the contribution of available nutrients and extracellular enzyme activity. In conclusion, it is essential to consider different soil layers when studying the effects of global climate change on the nutrient cycling of the biological soil crust.

## 1 Introduction

The freeze-thaw cycle (FTC) is an intricate series of events brought on by phase changes in soil moisture due to diurnal temperature fluctuations that result in variations in soil temperatures above and below 0°C. It is a relatively common natural phenomenon in temperate, mid-latitude, and high-altitude ecosystems during winter and spring (George et al., 2021). Seasonal FTC affects about 55% of the Northern Hemisphere’s land area, and the variability of FTC patterns (intensity, frequency, duration, etc.) depends mainly on the local climatic conditions (Gao et al., 2021a). Over the past 120 years, climate change has prompted a 1.59°C increase in land surface temperature, particularly in temperate desert regions where the warming effect is most pronounced (IPCC, 2021). Temperate deserts are extremely arid and mono-biodiverse and are among the typical global ecologically fragile areas sensitive to climate change (Li et al., 2021a). Scientists have predicted that global warming will reduce the volume and duration of snow cover and increase the frequency of FTC in deserts over the next 30 years (Mellander et al., 2007; Ji et al., 2014). Snow acts as an insulating layer, and its absence exposes the soil to diurnal temperature variations, increasing the intensity, frequency, and duration of FTC (Bokhorst et al., 2013). There is growing evidence that these changes in FTC patterns can affect the soil ecosystem. FTCs alter soil structure (Zhang et al., 2016; Zhang et al., 2021), water cycling (Wang et al., 2021; Li et al., 2021b), gas emission (Hamamoto et al., 2020; Yang et al., 2022a), and litter decomposition (Pelster et al., 2013). Studies have also shown that FTCs affect microbial community structure and function (Liu et al., 2021; Sang et al., 2021) and plant root dynamics (Tierney et al., 2001; Sorensen et al., 2016).

FTC impacts soil nutrient cycling through both physical and biological mechanisms. Through repeated expansion and contraction of water in the soil, FTCs physically break down soil aggregates and humus and increase soil nutrient content (Oztas and Fayetorbay, 2003; Hui et al., 2022). Soil particle fragmentation increases soil porosity and surface area (Ma et al., 2019), improving the sorption capacity of soil microorganisms and plant roots while increasing the risk of nutrient leaching (Campbell et al., 2014; Zhang et al., 2016). Moreover, ice crystals can kill or dissolve microorganisms and release cell contents, especially monosaccharides and amino acids, in the early stage of FTC (Song et al., 2017; Gao et al., 2021a). Freezing decreases the activity and effectiveness of soil microorganisms and plant roots in utilizing nutrients, resulting in soil enrichment (Tierney et al., 2001; Sorensen et al., 2018). At the same time, changes in soil nutrients further affect nutrient utilization and vegetation growth in the post-thaw growing season, causing continuous effects on ecosystem structure and function (Urakawa et al., 2014). Ice crystals in soil trap denature soil extracellular enzymes and limit their activity (Miura et al., 2019). From the perspective of extracellular enzymes, their ability to respond rapidly to changes in the soil environment is considered a good indicator for studying biochemical processes and nutrient limitations in soil ecosystems (Allison et al., 2007). The results of research on the impact of FTC on soil extracellular enzyme activity, however, have been inconsistent, mainly promoting (Sistla and Schimel, 2013; Okonkwo et al., 2022; Yang et al., 2022b), inhibiting (Tan et al., 2014; Hamamoto et al., 2020; Li et al., 2020), or having no discernible impact (Miura et al., 2019). This may be due to differences in soil substrate concentration, soil pH, and response to environmental changes corresponding to the different enzymes. Recently, many studies have focused on how FTC affects soil nutrient cycling in the tundra, plateau, forest, grassland, wetland, and farmland ecosystems, but research for desert ecosystems is lacking (Yu et al., 2011; Gao et al., 2021b).

Water is the limiting factor for ecosystems in temperate desert regions where rainfall is insufficient and cannot support the distribution of large areas of vascular plants, creating a distinctive landscape of drought-tolerant shrubs and biological soil crusts (Zhang et al., 2007). Biological soil crusts, which are common in arid and semi-arid regions and can sometimes cover up to 70% of the surface, are complexes made up of algae, lichens, mosses, and other soil microorganisms (Belnap et al., 2008; Rodriguez-Caballero et al., 2018; Weber et al., 2022). As the ‘engineers’ of ecosystems, they play crucial ecological roles in promoting the establishment of vascular plants, stabilizing soils, regulating hydrological processes, and cycling nutrients (Bowker et al., 2018; Xiao et al., 2022). In contrast to many vascular plants of cold temperate drylands, biological soil crusts are more active during winter snowpack and spring freeze-thaw periods, particularly during the latter when they can store up to 49% of the year’s carbon (Su et al., 2013; Yin and Zhang, 2016). Therefore, biological soil crusts can withstand low temperatures, and the freeze-thaw period is critical for their growth. However, previous studies have mainly focused on soil carbon, nitrogen, and phosphorus levels during changes in winter snow accumulation and not on the response pattern of biological soil crusts to freeze-thaw cycles and their role in nutrient cycling.

In summary, we pose the following scientific question: how does the frequency of freeze-thaw cycles affect carbon, nitrogen, and phosphorus cycling in desert moss crust soil, and does this effect vary between different soil layers? In light of these issues, we put forward the following scientific hypothesis: (1) Fragmentation of soil aggregates, structural changes, and cell lysis of microorganisms and plant roots by FTCs promote the uptake of soil nutrients such as carbon, nitrogen, phosphorus, and the corresponding enzyme activities. (2) As a result of the insulating properties of the moss crust, the lower soil temperature is comparatively stable, and the lower layer of the crust is less affected by freeze-thaw cycles than the crust layer is. This study chose the typical temperate desert Gurbantunggut as the study area and the moss crust at an advanced stage of biological soil crust development as the research object to test the above scientific hypotheses. By simulating different numbers of freeze-thaw cycles (0, 5, and 15), we investigated the impact of freeze-thaw cycles on nutrient cycling in the crust layer and the 0-3 cm soil layer below the crust. We also analyzed the key factors and pathways affecting the change of soil nutrient multifunctionality in different soil layers.

## 2 Materials and methods

### 2.1 Study area

Gurbantunggut Desert, the largest fixed and semi-fixed desert in China, is situated in the hinterland of the Junggar Basin in northern Xinjiang (44.18°-46.33° N, 84.52°-90.00° E). Here, the average annual precipitation is less than 150 mm, and annual evaporation exceeds 2000 mm. The average annual temperature is 6–10°C with extremes of 40°C or more, the annual cumulative temperature of 3000–5000°C in years ≥10°C, and average relative humidity of 50%-60%, with May through August typically below 45% (Zhang et al., 2007). The basic landscape features of the desert are linear and dendritic longitudinal dunes, and the vegetation is a shrub and small tree communities consisting of *Haloxylon persicum*, *Haloxylon ammodendron*, *Ephedra przewalskii*, *Calligonum mongolicum*, and other sandy plants (Zhang, 2020). Unlike other desert ecosystems, the Gurbantunggut Desert has a stable snow cover of 15–30 cm in winter, which accounts for 25% of annual precipitation and provides a suitable environment for the development of biological soil crusts in this desert. Depending on the predominant taxa, biological soil crusts are of three types: algal, lichen, and moss crusts. Moss crusts indicate an advanced stage of succession, and *Syntrichia caninervis* is the predominant species.

### 2.2 Experimental design

Samplings were conducted in September 2019. We created a 50 m × 50 m sample plot in the interhall lowlands of the hinterland of the Gurbantunggut Desert and selected well-developed and uniform habitat patches of *Syntrichia caninervis*. Undisturbed columnar soil profiles were then collected using homemade PVC pipes (10 cm in diameter and 15 cm in height). Before sampling, the humus on the surface of the crust was removed, *Syntrichia caninervis* was sprayed wet, and the PVC pipe was inserted vertically to prevent further harm to the structural integrity of the crust and soil. They were carefully removed, sealed with nylon mesh, and returned to the laboratory. Before processing, all collected samples were dried for 10 days at a moderate temperature (25°C) in a sunroom to prevent differences in water content and other factors.

The finished samples were randomly divided into three groups for simulated FTC experiments in a constant-temperature incubator. Based on the water equivalent of the natural winter snowpack in Gurbantunggut Desert, a snowpack equivalent to 15 mm of rainfall was added to each sample separately to simulate stable snow events in winter. A comparative experiment with fewer FTCs was designed in light of the sensitive response of desert regions to ongoing global warming, particularly the higher warming in winter than in summer (IPCC, 2021). For each group, three treatments of 0, 5, and 15 FTCs were performed with five replicates each. Each FTC consisted of 24 h: 12 h freezing at –10°C and 12 h thawing at 10°C. After incubation, the temperatures of the 0 and 5 FTCs were raised to 10°C and maintained there for 15 days (Yin et al., 2021). After the completion of experiments, the moss crust was peeled off, and 30 soil samples were collected using a ring knife to scrape the soil from beneath the crust’s 0–3 cm layer and filter it through a 2-mm sieve. Each soil sample was divided into two portions, placed in the freezer at –20°C, and allowed to dry naturally before testing.

### 2.3 Analysis of the physical and chemical properties of the soil

Fresh soil was dried to constant weight at 105°C for 48 h and then weighed to determine soil water content (SWC). The pH was determined by the potentiometric method at a water-soil ratio of 1:2.5. Electrical conductivity (EC) was determined using the AC conductivity method at a water-soil ratio of 1:5 (Bhattacharyya et al., 2021). Total carbon (TC) and organic carbon content (SOC) in soil were determined using a carbon and nitrogen analyzer (Multi 3100C/N, Analytik Jena AG, Germany) by the combustion method and HCL titration-combustion method, respectively. The nutrient levels in the soil were measured using a fully automatic flow-through analyzer (Bran Luebbe, AA3, Germany): (1) total nitrogen (TN) and total phosphorus (TP) after ablation with concentrated sulfuric acid, perchloric acid, and hydrofluoric acids; (2) ammonium nitrogen (NH_4_^+^-N) and nitrate nitrogen (NO_3_^–^N) after leaching with 0.01 mol/L CaCl_2_ solution; and (3) available phosphorus (AP) after leaching with a 0.5 mol/L NaHCO_3_ solution. We also estimated total nitrogen by the Kjeldahl method, ammonium nitrogen by indophenol blue colorimetry, nitrate nitrogen by phenol disulfonic acid colorimetry, and total and available phosphorus by molybdenum antimony anti-colorimetric method (Edwards and Jefferies, 2013).

### 2.4 Analysis of extracellular enzyme activity

We analyzed soil enzymes associated with the carbon, nitrogen, and phosphorus cycles. Enzyme activities were estimated colorimetrically at different wavelengths (see below):

Carbon cycle: β-glucosidase (BG), which catalyzes p-nitrophenyl-β-D-glucopyranoside to form p-nitrophenol (PNP), was measured colorimetrically at 405 mm. Sucrase (SR), which catalyzes the reaction of sucrose with 3,5-dinitrosalicylate to form a colored compound, was measured colorimetrically at 540 nm. Peroxidase (POD) and Polyphenol oxidase (PPO), which catalyze the conversion of gallic acid to purple gallic acid, were measured colorimetrically at 430 nm.

Nitrogen cycle: Soil urease (UA), which catalyzes the reaction of urea with hypochlorite and phenol to form indophenol blue, was measured colorimetrically at 578 nm. Nitrate reductase (NR), which catalyzes the conversion of nitrate in the soil to nitrite, was measured colorimetrically at 540 nm.

Phosphorus cycle: Alkaline phosphatase (AKP), which catalyzes the formation of yellow PNP products from phosphoric acid to nitrate, was measured colorimetrically at 405 nm. Phytase, which hydrolyzes sodium phytate to produce inorganic phosphorus, was measured colorimetrically at 700 nm.

### 2.5 Statistical analysis

All data were tested for normality and chi-square using SPSS 19.0 software, and a two-factor ANOVA was performed for FTC, soil layer, and the interaction between the two. One-way ANOVA was used to analyze the nutrient levels of carbon, nitrogen, and phosphorus and the associated enzyme activities for different FTC frequencies and soil layers. Significant differences between treatments were determined using Tukey’s test and plotted using Origin 2018 software.

Soil multifunctionality (SMF) is a comprehensive index for evaluating the ability of soils to maintain multiple ecological functions simultaneously, and the most common calculation methods include the mean method, multiple threshold method, single function method, and single threshold method. The mean method is widely used in multifunctionality studies and provides an intuitive and easily interpreted measure to better assess the ability of soils to maintain multiple functions (Maestre et al., 2012; Byrnes et al., 2014). This study used the mean method to calculate 15 indicators of soil nutrient ecological functions, encompassing the carbon cycle, nitrogen cycle, and phosphorus cycle. The Z-score was used to standardize the data, and the soil nutrient multifunctionality index was determined by averaging all the indicators, which ensured that the data were on the same scale.

The “innerplot” function of the “plspm” package was used in R 4.2.1 to investigate the direct and indirect relationships between the number of FTCs, soil water content, pH, electrical conductivity, total nutrients, available nutrients, extracellular enzyme activity, and soil nutrient multifunctionality. Before modeling, the “varclus” function of the “Hmisc” package was used to remove redundancy and multicollinearity from the ANOVA. Total nutrients (TC, SOC, TN, TP), available nutrients (NH_4_^+^-N, NO_3_^–^N, AP), and extracellular enzyme activities (BG, POD, SR, PPO, UA, NR, AKP, phytase) in the model set were determined by multiple regression. This model was designed to explore the effects of direct or indirect pathways of each factor and to further identify the key factors affecting changes in soil nutrient multifunctionality and their possible influence pathways.

## 3 Results

### 3.1 Physicochemical properties of soil

**Figure 1:**
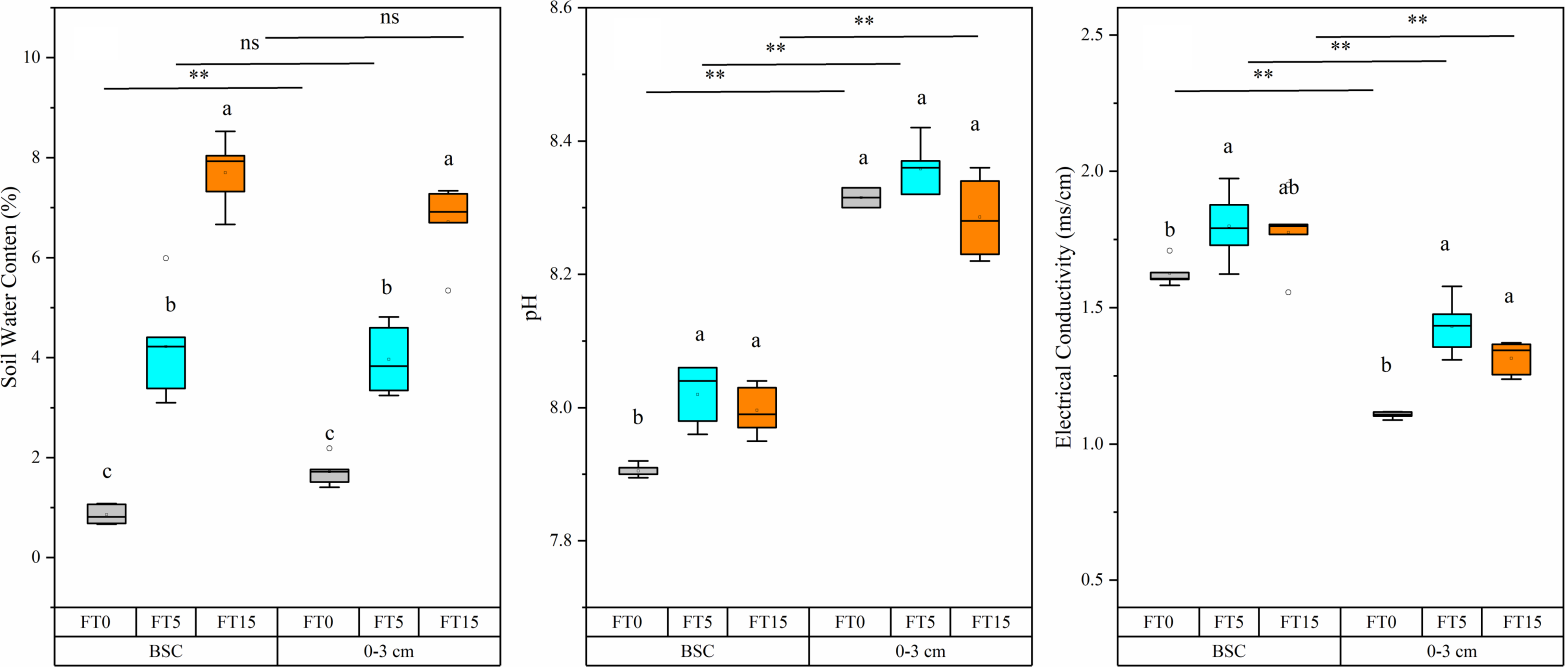
Effect of FTC on water content, pH, and electrical conductivity in different soil layers. Results are mean ± SE of five independent replicates. Different lowercase and uppercase letters indicate significant differences between the FTC treatments (P < 0.05), **(P < 0.01), and ns (P > 0.05).

FTC, soil layer, and their interaction significantly altered the water content, pH, and conductivity of moss crust soil (P < 0.05, **Table 1**, **Figure 1**). The water content of the soil increased gradually with an increase in the FTC number. The pH and electrical conductivity first increased and then decreased after five FTCs. In the different soil layers, pH increased significantly while electrical conductivity decreased significantly at different freeze-thaw frequencies in the 0–3 cm layer compared to the crust layer. However, water content increased significantly only in the absence of freeze-thaw.

**Table 1:** Two-way analysis of variance for physicochemical properties of the soil.

Indexes	Freeze-thaw cycles	Layer	Freeze-thaw cycles × Layer
SWC	169.623**	0.233	4.150*
pH	9.795**	555.097**	5.638**
EC	17.602**	162.724**	1.556
TC	19.092**	207.609**	0.879
TN	0.916	36.174**	0.318
TP	8.728**	27.976**	3.283
SOC	16.992**	179.731**	8.153**
NH_4_^+^	33.157**	65.465**	15.172**
NO_3_^-^	25.802**	77.909**	2.008
AP	144.311**	7.551*	27.905**
BG	57.424**	147.032**	200.094**
PPO	32.395**	222.355**	77.730**
SR	1.262	305.909**	10.446**
POD	7.873**	31.379**	3.570*
UA	16.218**	37.852**	3.171
NR	36.048**	39.835**	30.790**
AKP	89.565**	352.466**	33.164**
Phytase	35.856**	1156.464**	1763.406**
SMF	75.406**	1197.709**	72.211**

* and ** denote P < 0.05 and P < 0.01, respectively. SWC, soil water contents; EC, electrical conductivity; SOC, soil organic carbon; TN, total nitrogen; TP, total phosphorus; NH_4_^+^, ammonium nitrogen; NO_3_^–^, nitrate nitrogen; AP, available phosphorus; BG, β-1,4-glucosidase; PPO, Polyphenol oxidase; SR, Sucrase; POD, Peroxidase; UA, Urease; NR, Nitrate reductase; AKP, Alkaline phosphatase.

### 3.2 Soil nutrient variation

**Figure 2:**
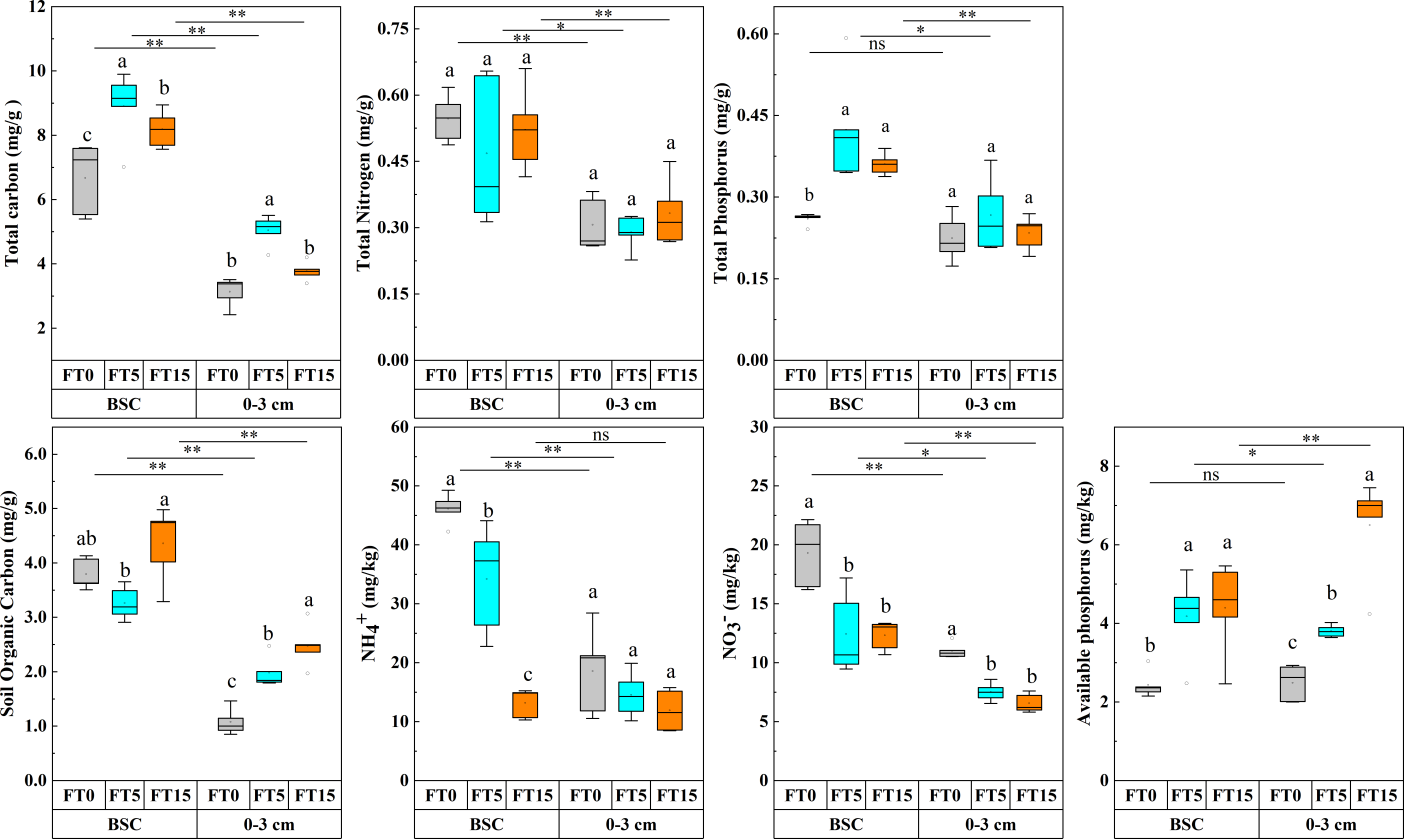
Effect of FTC on soil nutrients in different soil layers. Results are mean ± SE of five independent replicates. Different lowercase and uppercase letters indicate significant differences between the FTC treatments (P < 0.05), *(P < 0.05), **(P < 0.01), and ns (P > 0.05).

Two-way ANOVA analysis showed that FTC and soil depth significantly affected the nutrient levels, namely, total carbon, total nitrogen, total phosphorus, organic carbon, nitrate-nitrogen, ammonium-nitrogen, and available phosphorus in the soil (P < 0.05, **Figure 2**). The interaction between the two factors had significant effects only on organic carbon, ammonium-nitrogen, and available phosphorus (P < 0.01, **Table 1**). As the number of FTCs increased, total carbon and phosphorus in different soil layers showed an increasing trend followed by a decreasing trend, nitrate and ammonium-nitrogen showed a decreasing trend, and available phosphorus showed an increasing trend. However, organic carbon first decreased and then increased in the crust layer while gradually increasing in the 0–3 cm layer. Except for total phosphorus and available phosphorus, all nutrient indicators of carbon, nitrogen, and phosphorus were significantly higher in the crust layer than in the 0–3 cm layer (P < 0.05, **Figure 2**).

### 3.3 Changes in the activity of extracellular enzymes in soil

**Figure 3:**
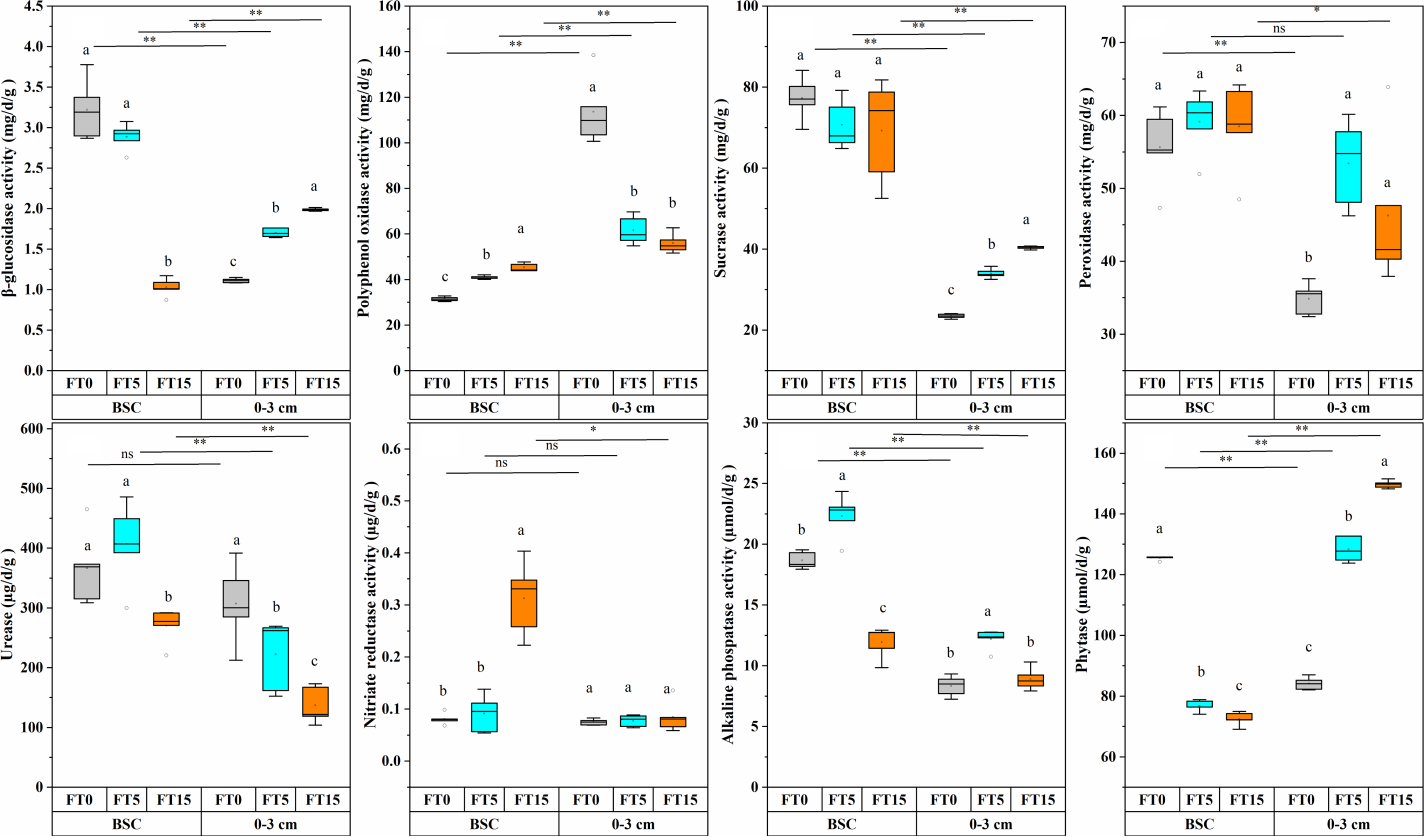
Effect of FTC on soil enzyme activity in different soil layers. Results are mean ± SE of five independent replicates. Different lowercase and uppercase letters indicate significant differences between the FTC treatments (P < 0.05), *(P < 0.05), ** P < 0.01), and ns (P > 0.05).

The activities of β-glucosidase, polyphenol oxidase, sucrase, peroxidase, urease, nitrate reductase, alkaline phosphatase, and phytase were significantly altered by FTC, soil layer, and their interaction (P < 0.01, **Table 1**, **Figure 3**). FTC had no discernible effect on sucrase, and neither soil layers nor freeze-thaw interaction had any effect on urease activity. As the number of freeze-thaws increased in the different soil layers, the trends of every soil enzyme indicator related to the carbon, nitrogen, and phosphorus cycles also started varying considerably. In the crust layer, β-glucosidase, sucrose, and phytase showed a decreasing trend; polyphenol oxidase, peroxidase, and nitrate reductase showed an increasing trend; and urease and alkaline phosphatase showed an increasing trend, followed by a decreasing trend (**Figure 3**). In the 0–3 cm layer, β-glucosidase, sucrose, and phytase showed an increasing trend; polyphenol oxidase, urease, and phytase showed a decreasing trend; and peroxidase and alkaline phosphatase showed an increasing trend followed by a decreasing trend (**Figure 3**). Compared to the crust layer, the activities of all soil enzyme indicators were significantly decreased in the 0–3 cm layer.

### 3.4 Soil multifunctionality and factors affecting its variability

**Figure 4:**
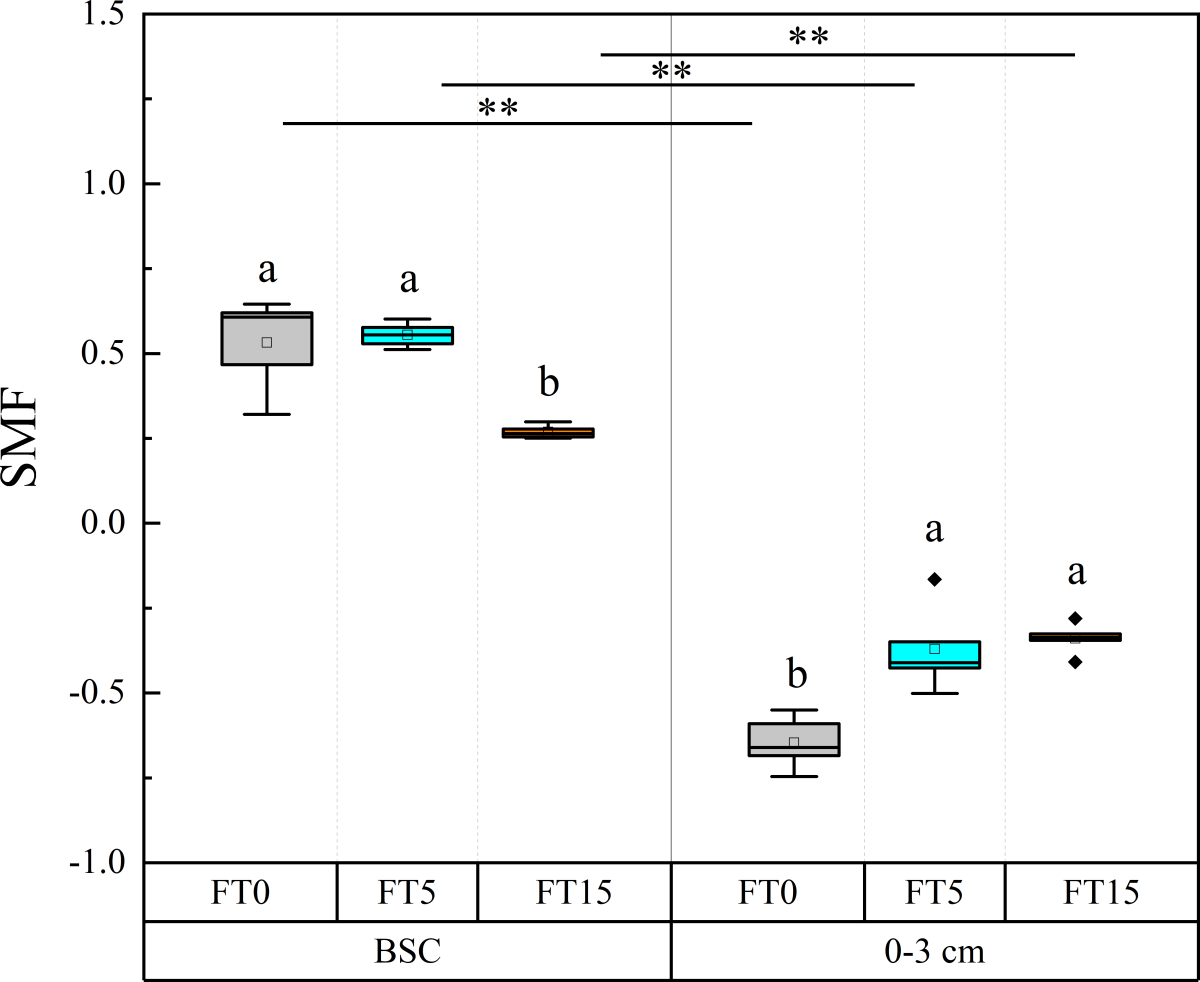
Effect of FTC on soil nutrient multifunctionality in different soil layers. Results are mean ± SE of five independent replicates. Different lowercase and uppercase letters indicate significant differences between the FTC treatments (P < 0.05), **(P < 0.01).

Two-factor ANOVA showed that FTC, soil depth, and the interaction of the two factors significantly affected soil nutrient multifunctionality (P < 0.01, **Table 1**, **Figure 4**). The trends of soil nutrient multifunctionality were opposite in different layers of soil. As the number of FTCs increased, soil nutrient multifunctionality decreased in the crust layer while it increased in the 0–3 cm layer. Overall, soil nutrient multifunctionality decreased with decreasing soil depth.

**Figure 5:**
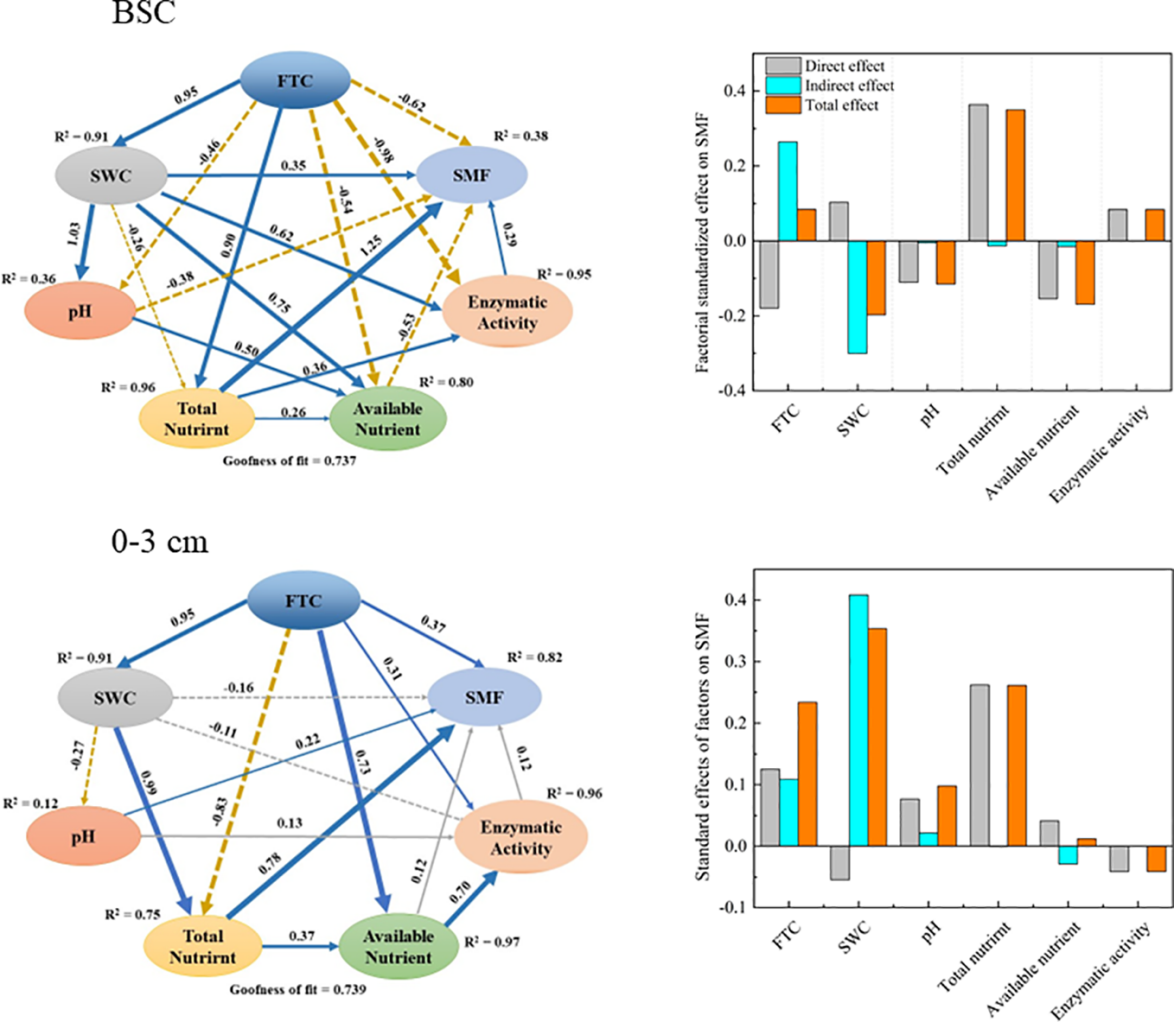
Partial least squares pathway model (PLS-PM) of soil nutrient multifunctionality (SMF) and environmental factors. The model illustrates the effects and pathways of FTC, soil water content (SWC), pH, total nutrients, available nutrients, and extracellular enzyme activity on soil nutrient multifunctionality. The blue solid and brown dashed arrows depict the direct positive and negative effects of causality (P < 0.05), and the gray solid and dashed arrows show the direct positive and negative effects of causality (P > 0.05), respectively.

Results from the PLS-PM model revealed that the factors in the crust layer factors explained 38% of the variation in SMF (**Figure 5**). The total amount of nutrients in the soil had a direct positive effect on SMF. Despite the fact that soil water content directly affected SMF negatively, it still had a significant positive effect on SMF indirectly through its effects on pH and total nutrients. In addition, available nutrients and pH both had a significant detrimental impact on SMF. Overall, total nutrients, soil water content, and available nutrients were the three most important factors affecting SMF in the crust layer.

In contrast, factors at the 0–3 cm layer explained 82% of the variation in SMF (**Figure 5**). The largest indirect and overall positive impact on SMF was provided by soil water content. Total nutrients and FTC also had strong positive effects on SMF. Overall, soil water content, total nutrients, and FTC were the three most important factors affecting SMF changes in the 0–3 cm layer.

## 4 Discussion

### 4.1 Effect of FTC on soil nutrients

The results partially confirmed our scientific hypothesis 1 that increasing FTC significantly increased the total carbon, organic carbon, total phosphorus, and available phosphorus content of moss crust soils, with the increase in available phosphorus content being particularly significant. There are several main reasons for the increase in soil carbon and phosphorus. First, the drastic physical effects of FTC significantly altered soil structure (Zhang et al., 2016). The repeated expansion and contraction of water caused by FTCs leads to the fragmentation of coarse-grained soils, large agglomerates, and organic and inorganic colloids and promotes the reduction of soil grain size (Oztas and Fayetorbay, 2003). Compared with coarse particles, the increase of fine soil particles promotes the accumulation and concentration of available phosphorus in the soil (Qian et al., 2013; Xiao et al., 2019; Shen et al., 2020). Second, the high concentrations of organophosphate and polyphosphate in soil microbial cells (Makarov et al., 2002) and ice crystals produced during FTC can perforate microbial cells and result in the accumulation of carbon and phosphorus nutrients (Larsen et al., 2002). Finally, FTC increased the mortality of overwintering plant roots and the input of plant apoplast, especially moss crusts and pseudoroots, which weakens the fixation of soil nutrients by plants and further promotes the accumulation of organic matter and nutrients (Tierney et al., 2001).

Contrary to the initial hypothesis, many previous studies suggested that increased FTC could promote higher soil nitrogen accumulation and efficacy (Christopher et al., 2008; Song et al., 2017; Gao et al., 2018). However, the changes in N content in this study were the opposite, with ammonium and nitrate content decreasing with increasing FTCs, which might be related to the increased physiological activity of moss crusts under FTCs (Su et al., 2013; Yin and Zhang, 2016). In a study by Yin et al. (2021) that examined the effects of FTCs on the physiological activity of moss crusts, it was discovered that inorganic nitrogen was the only direct nitrogen source required for moss crust growth, and the physiological activity of moss crusts increased with increasing FTCs. The activity of moss crusts and their symbiotic nitrogen-fixing microorganisms may also be limited in the early stages of FTC with reduced nitrogen fixation (Wang et al., 2013). Moreover, the emission and leaching of nitrogenous gases such as N_2_O is also important reason for the decrease in inorganic nitrogen. According to previous studies, water freezing prevents oxygen diffusion and makes soils susceptible to an anaerobic environment, which promotes denitrification and accelerates soil nitrogen loss (Teepe et al., 2001; Pelster et al., 2019). Water infiltration brought on by snowmelt also increases the risk of inorganic nitrogen loss, causing a further decrease in the inorganic nitrogen content. In addition, total carbon, total nitrogen, total phosphorus, nitrate nitrogen, and available phosphorus content of the soil stabilized after 15 FTCs. It suggests a time lag in the adaptive mechanisms of soil microorganisms in the face of disturbances to the soil environment by FTCs (Jansson and Hofmockel, 2020). In a study of biological soil crusts in the Kubuqi Desert, Wang et al. (2015b) found similar results. The total carbon and total nitrogen contents of the algal crust in the soil reached an equilibrium after 4–5 days of FTCs, which may be related to the decrease in microbially available substrates.

The soil carbon, nitrogen, and phosphorus-related nutrient contents in the subcrustal layer were significantly lower than in the crust. In addition, FTC had a higher impact on nutrients in the crust. The crust layer is exposed to the soil surface and mediates snow melting, making it vulnerable to FTCs. In addition to the physical effect of FTC, soil nutrients in the crust layer were also influenced by the biological effect of the moss crust itself. The findings of this study revealed that the crust layer was the only one where FTC had an impact on the contents of total nitrogen, total phosphorus, and ammonium nitrogen; the three did not change significantly as the number of FTC treatments increased in the 0–3 cm soil layer. It may be related to the insulating effect of moss crusts, which reflect only half of the surface light compared with bare sand or cyanobacterial crusts, thus reducing the surface energy flux and increasing the surface temperature (Belnap, 1995). Even in cold winter, moss crusts can increase soil temperature in the 0–5 cm layer by about 8°C (Xiao et al., 2016). However, studies in exposed sands of desert ecosystems have revealed that FTC significantly affects soil nutrients in the top 10 to 20 cm of soil (Zhao et al., 2008; Hu et al., 2015). It may imply that moss crusts can mitigate the disturbance of soil nutrients by FTC (Wang et al., 2015b). In addition, the available phosphorus content did not vary significantly with soil depth but increased significantly in the 0–3 cm layer with increasing FTCs relative to the crust, which could be due to leaching and loss of available phosphorus due to the downward migration of water after snowmelt (Shen et al., 2020).

### 4.2 Effect of FTC on soil extracellular enzyme activity

Soil extracellular enzymes mediate many biochemical processes in soil, such as organic matter decomposition, nutrient cycling, and energy flow, and are considered sensitive indicators of ecosystem function (Yao et al., 2006; Ghiloufi et al., 2019). The main sources of soil secretions are microorganisms, plant roots, and soil animals (Ghiloufi et al., 2019), while a variety of biotic and abiotic factors, such as moisture, temperature, and nutrient content, influence soil enzyme activity (Sardans et al., 2008). As a result, the FTC-induced change in the soil’s temperature and moisture level significantly impacts the activity of soil enzymes (Miura et al., 2019). In this study, the decomposition and transformation processes of various soil nutrients of moss crust were examined in the context of selected hydrolases and oxidases associated with the carbon, nitrogen, and phosphorus cycle. The results refuted the initial hypothesis 1 that FTC inhibits the activities of soil enzymes, with the exception of carbon cycle-related oxidases and nitrate reductases. However, the results between the different soil layers essentially confirmed hypothesis 2 that the insulating effect of the moss crust provides a suitable environment for the increase of extracellular enzyme activities in the soil below the crust.

Freeze-thaws had a significant impact on the carbon cycle enzymes’ activities. In the crust, FTCs decreased hydrolase activity (β-glucosidase and sucrase) but increased oxidase activity (peroxidase and polyphenol oxidase). However, in the 0–3 cm layer, carbon cycle-related enzymes showed an opposite trend. β-Glucosidase and sucrase hydrolyze disaccharides into monosaccharides for plant uptake and are essential for carbon cycling. With the increase of FTCs, β-glucosidase and sucrase showed a decreasing trend in the crust layer. Consistent with previous studies, FTC altered soil microbial structure and function, and reduced microbial biomass and hydrolase activity (Sorensen et al., 2018; Li et al., 2020; Liu et al., 2021; Gao et al., 2021a). In addition, low soil temperatures, reduced water availability, and depletion of dead microbial substrates may limit microbial and enzymatic activities, thereby affecting carbon decomposition by soil microorganisms (Koponen et al., 2006; Sorensen et al., 2018; Wang et al., 2021). However, peroxidase and polyphenol oxidase, involved in the oxidation and degradation of reactive lignin, cellulose groups, and phenolics (Burke and Cairney, 2002; Toberman et al., 2008), showed an increasing trend in the crust layer with increasing FTCs. Peroxidase and polyphenol oxidase are produced in greater amounts in the crust layer as a result of the buildup of difficult-to-degrade materials like plant roots, apoplastic material, and moss pseudoroots due to FTCs (Pind et al., 1994; Bai et al., 2021). This may account for the increased activity of these two enzymes. Remarkably, β-glucosidase, sucrose, and peroxidase showed opposite trends in the 0–3 cm layer compared to the crust layer. The environmental differences in the different soil layers of the moss crusts affected the changes in soil enzymes during the freeze-thaw period. Unlike sandy areas, the darker color and dense structure of moss crusts blocked the direct effects of snow and strong wind on the soil and maintained relatively high soil temperatures (Xiao et al., 2016). Several studies have confirmed the temperature dependence of extracellular soil enzymes (Lang et al., 2000; Sistla and Schimel, 2013; Okonkwo et al., 2022) and the significant increase of β-glucosidase and sucrase in the 0–3 cm layer might be due to the early thawing of the soil (Bell et al., 2010). In addition, peroxidases in the 0–3 cm layer decreased rapidly after the onset of FTC, probably because low temperatures inhibited their activity (Freeman et al., 2001). This suggests that the hydrolases of the carbon cycle have a higher cold tolerance and sensitivity to temperature fluctuations compared to the oxidases.

FTC significantly inhibited urease activity in the soil nitrogen cycle and promoted nitrate reductase activity, resulting in soil nitrogen loss. Urease and nitrate reductase in soil mediate the conversion process between organic, ammonium, and nitrate nitrogen, thus influencing soil nitrogen accumulation and effectiveness (Zornoza et al., 2006; Wang et al., 2015a). In the desert moss crust, decreased soil urease activity and increased nitrate reductase activity with increasing FTC resulted in a significant decrease in ammonium and nitrate nitrogen. Previous studies have also reported negative effects of FTC on urease activity, with similar trends for ammonium nitrogen (Miura et al., 2019; Hou et al., 2020). The low temperature might inhibit the decrease in urease activity (Cao et al., 2003). In addition, the increase in nitrate reductase is closely linked to the enhancement of denitrification. Studies on the effects of FTC on N_2_O emissions have shown that freeze-thaw increases the expression of denitrification genes and their microbial activity, thereby enhancing the release of N_2_O gas (Muller et al., 2003; Sharma et al., 2006). One of the mechanisms is that the denitrification process and the soil oxygen content have a negative correlation, and the gradual increase in water content following the thawing of snow cover in the soil decreases the availability of oxygen (Morkved et al., 2006). Moreover, the ice layer that forms after surface freezing blocks oxygen exchange, intensifying the anaerobic environment of the soil and boosting the activity of the microbial organisms involved in the denitrification process, which in turn stimulates nitrate reductase activity (Uchida and Clough, 2015).

Due to FTCs, the activities of soil enzymes related to phosphorus cycling were inhibited in the crust layer and promoted in the 0–3 cm layer. Phosphatases and phytases catalyze the mineralization process of organic phosphorus in soils; thus, their activity levels directly affect soil phosphorus efficacy (Turner et al., 2002; Condron et al., 2005; Rocky-Salimi et al., 2016). Since much of the phosphorus in soil is organically bound but unavailable to plants, the mineralization of organic phosphorus by phosphatases and phytases directly affects nutrient availability for plants and microorganisms (Nannipieri et al., 2002). With increasing FTC, alkaline phosphatase decreased along with phytase in the crust and increased in the 0–3 cm layer. Studies have shown that the structure of the soil, temperature, pH, and substrate content and type affect the activity of alkaline phosphatase and phytase (Staddon et al., 1998; Vinjamoori et al., 2004). Their decrease in the crust layer contrasts with the increase in available phosphorus because the accumulation of available phosphorus may reduce the demand of soil microorganisms for elemental phosphorus hydrolases (Bai et al., 2021). The lower temperature of the crust layer and the competition between mosses and microorganisms for nutrients may also decrease alkaline phosphatase and phytase activities (Champion et al., 2000; Terefe et al., 2004; Azeem et al., 2015). In addition, the changes in alkaline phosphatase and phytase were not identical, and we found a significant increase in alkaline phosphatase after 5 FTCs, which could be due to the availability of sufficient substrate and a more suitable pH environment for alkaline phosphatase in the early FTC soils (Dick et al., 2000). The significant increase in phytase activity in the 0–3 cm layer was also more consistent with the change in available phosphorus, which may indicate that phytase is more important than phosphatase for the sequestration of available phosphorus in the soil during the freeze-thaw period in desert.

### 4.3 Effect of FTC on the multifunctionality of soil nutrients

In general agreement with scientific hypothesis 2, the variability of the different FTC frequencies affected the changes in soil nutrient multifunctionality in the different soil layers of the moss crusts. Specifically, with the increase in FTC frequencies, the soil nutrient multifunctionality in the crust layer decreased significantly after fifteen cycles, while it increased significantly in the 0–3 cm layer after five cycles. This indicates the negative effect of heavy FTC (15 cycles) on the soil environment in the crust layer, while light FTC (5 cycles) promoted the soil function of the lower layer. Similar outcomes were obtained by Liu et al. (2022) for 0–7.5 cm soils in temperate forest ecosystems at the same latitude in China, where the soil multifunctional index gradually increased before FTC treatment, peaked at seven cycles, and then significantly decreased. However, the thickness of the naturally removed moss crust layer was only about 1.5–2.5 cm (Belnap, 2003b). In this study, the change in nutrient multifunctionality reversed only in soils 3 cm below the crust layer and increased significantly in the early stage of FTC. The above results indicate the potential role of moss crusts in maintaining the stability of nutrient multifunctionality in the topsoil. The insulating effect of moss crust regulates several ecosystem processes and functions, such as water evaporation, microbial activity, nutrient cycling efficiency, and plant nutrient availability (Belnap, 2003a). In addition, the resistance of moss crusts to weathering erosion and their physiological-ecological adaptations during FTC complicates the multifunctional changes in soil nutrients (Zhang et al., 2010; Yin et al., 2021; Zhang et al., 2022).

The response of soil nutrient multifunctionality to various factors of FTC regulation was also different in different soil layers. Results from the SEM model results showed contrasting effects of soil water content, pH, available nutrients, and extracellular enzyme activity on nutrient multifunctionality in different soil layers. Soil water content was the biggest limiting factor in the crust layer, while in the 0–3 cm layer, it positively affected nutrient multifunctionality. It suggests that in addition to adequate moisture, water availability during the freeze-thaw period is also critical (Hui et al., 2022). Compared to the lower layer, the exposed crust layer froze first and thawed relatively late in the cold, which limited microbial and plant root activity and reduced water availability (Man et al., 2019). FTC had an indirect positive effect on nutrient multifunctionality in both the soil layers, but the direct effect was reversed, reducing the overall effect of FTC in the crust layer. FTC causes soil structure reorganization, agglomerate fragmentation, temperature fluctuations, and recurrent phase changes of water, all of which increased water availability, nutrient accumulation, and nutrient redistribution (Oztas and Fayetorbay, 2003; Cornelissen et al., 2007; Song et al., 2017; Sang et al., 2021; Wang et al., 2021). All these effects, in turn, indirectly result in changes in the multifunctionality of soil nutrients. The direct negative impact of the crust layer may result from the limitation of microbial activity due to low temperature, inhibition of the physiological activity of moss crust, and damage to the root system by prolonged FTC, thus negatively affecting the multifunctionality of the soil. In addition, total nutrients were the dominant factor in changes in soil nutrient multifunctionality in both the crust layer and the 0–3 cm layer, indicating that total nutrients are more significant in the soil functional environment than available nutrients and extracellular enzyme activity.

## 5 Conclusion

FTC significantly affected carbon-, nitrogen-, and phosphorus-related nutrients, extracellular enzyme activities, and nutrient multifunctionality in the soil. The results showed that FTC increased the levels of carbon and phosphorus-related nutrients and decreased the efficacy of nitrogen nutrients. However, soil nutrient changes gradually stabilized after 15 FTCs. Soil enzymes catalyzing the conversion of various carbon, nitrogen, and phosphorus were inhibited by frost, low temperature, and anaerobic conditions and showed a decreasing trend. Remarkably, the changes in extracellular enzymes mediating the degradation of various substances in the different soil layers of moss crusts varied greatly with increasing FTC and even showed opposite trends. It explains the complex effects of FTC-induced changes in the soil environment on various microbial functional groups and nutrient cycling processes. The multifunctionality of soil nutrients decreased in the crust layer and increased in the 0–3 cm layer due to changes in soil nutrients and enzyme activities. Water also significantly improved the multifunctionality of nutrients in the 0–3 cm layer compared to the crust layer. It may indicate that the higher soil temperature and water availability under the moss crust layer promote soil microbial activity and nutrient cycling and storage, suggesting a possible role for the moss crust in mitigating the negative effects of FTC on desert topsoil. The importance of biological soil crusts may eventually increase with climate changes related to global warming and changes in FTCs and patterns having even greater impacts on the soil nutrient environment.

## Data availability statement

The raw data supporting the conclusions of this article will be made available by the authors, without undue reservation.

## Author contributions

BFY, NW, XBZ, and YZ planned and designed the research. QZ analyzed data and wrote the manuscript. QZ, JL, SZ, and YL performed experiments. All authors contributed to the article and approved the submitted version.
